# High-throughput sequence alignment using Graphics Processing Units

**DOI:** 10.1186/1471-2105-8-474

**Published:** 2007-12-10

**Authors:** Michael C Schatz, Cole Trapnell, Arthur L Delcher, Amitabh Varshney

**Affiliations:** 1 Center for Bioinformatics and Computational Biology, University of Maryland, College Park, MD, USA; 2 Department of Computer Science, University of Maryland, College Park, MD, USA

## Abstract

**Background:**

The recent availability of new, less expensive high-throughput DNA sequencing technologies has yielded a dramatic increase in the volume of sequence data that must be analyzed. These data are being generated for several purposes, including genotyping, genome resequencing, metagenomics, and *de novo *genome assembly projects. Sequence alignment programs such as MUMmer have proven essential for analysis of these data, but researchers will need ever faster, high-throughput alignment tools running on inexpensive hardware to keep up with new sequence technologies.

**Results:**

This paper describes MUMmerGPU, an open-source high-throughput parallel pairwise local sequence alignment program that runs on commodity Graphics Processing Units (GPUs) in common workstations. MUMmerGPU uses the new Compute Unified Device Architecture (CUDA) from nVidia to align multiple query sequences against a single reference sequence stored as a suffix tree. By processing the queries in parallel on the highly parallel graphics card, MUMmerGPU achieves more than a 10-fold speedup over a serial CPU version of the sequence alignment kernel, and outperforms the exact alignment component of MUMmer on a high end CPU by 3.5-fold in total application time when aligning reads from recent sequencing projects using Solexa/Illumina, 454, and Sanger sequencing technologies.

**Conclusion:**

MUMmerGPU is a low cost, ultra-fast sequence alignment program designed to handle the increasing volume of data produced by new, high-throughput sequencing technologies. MUMmerGPU demonstrates that even memory-intensive applications can run significantly faster on the relatively low-cost GPU than on the CPU.

## Background

Sequence alignment has a long history in genomics research and continues to be a key component in the analysis of genes and genomes. Simply stated, sequence alignment algorithms find regions in one sequence, called here the query sequence, that are similar or identical to regions in another sequence, called the reference sequence. Such regions may represent genes, conserved regulatory regions, or any of a host of other sequence features. Alignment also plays a central role in *de novo *and comparative genome assembly [1,2], where thousands or millions of sequencing reads are aligned to each other or to a previously sequenced reference genome. New, inexpensive large-scale sequencing technologies [3] can now generate enormous amounts of sequence data in a very short time, enabling researchers to attempt genome sequencing projects on a much larger scale than previously. Aligning these sequence data using current algorithms will require very high-performance computers, of the type currently available only at the largest sequencing and bioinformatics centers. Furthermore, realizing the dream of widespread personal genomics at hospitals and other clinical settings requires sequence alignment to be low cost in addition to high-throughput.

Most personal computer workstations today contain hardware for 3D graphics acceleration called Graphics Processing Units (GPUs). Recently, GPUs have been harnessed for non-graphical, general purpose (GPGPU) applications. GPUs feature hardware optimized for simultaneously performing many independent floating-point arithmetic operations for displaying 3D models and other graphics tasks. Thus, GPGPU programming has been successful primarily in the scientific computing disciplines which involve a high level of numeric computation. However, other applications could be successful, provided those applications feature significant parallelism.

In this paper, we describe a GPGPU program called MUMmerGPU that performs exact sequence alignment using suffix trees on graphics hardware. Our implementation runs on recent hardware available from nVidia using a new software development kit (SDK) for GPGPU progamming called Compute Unified Device Architecture (CUDA). MUMmerGPU is targeted to tasks in which many small queries, such as reads from a sequencing project, are aligned to a large reference sequence. To assess the performance of MUMmerGPU we compare it to the exact alignment component of MUMmer called mummer. MUMmer is a very fast and widely used application for this type of task [4], and is also used as the alignment engine for the comparative assembler AMOScmp [2]. Overall MUMmerGPU is more than three times faster than mummer on typical sequence alignment tasks involving data from three recent sequencing projects. As implemented, MUMmerGPU is a direct replacement for mummer and can be used with any other programs that process mummer output, including the other components of MUMmer that post-process the exact alignments computed by mummer into larger inexact alignments.

### Sequence alignment

One of the most successful algorithms for computing alignments between sequences is MUMmer [4-6]. The first stage of MUMmer is performed by a component called mummer, which computes exact alignments between the pair of sequences. These alignments can be used directly to infer large-scale sequence structure, or they can be used to seed extensions to longer inexact alignments using the post-processing tools bundled with MUMmer. Unlike other popular sequence alignment programs such as BLAST [7], FASTA [8], and LAGAN [9], which use fixed length seeds for constructing their alignments, mummer alignments are variable-length maximal exact matches, where maximal means that they cannot be extended on either end without introducing a mismatch. First, mummer pre-processes the reference sequence to create a data structure, called a *suffix tree*. This data structure allows mummer to then compute all maximal exact substring alignments of a query sequence in time proportional to the length of the query. The time to pre-process the reference sequence is proportional to its length (which may be considerable for very long sequences), but this time becomes insignificant when amortized across many query searches. Consequently, suffix trees are used in several alignment algorithms, including MGA [10] and REPuter [11]. The suffix tree [12] for string *S *is a tree that encodes every suffix of *S *with a unique path from the root to a leaf. For a string of length *n*, there are *n *leaf nodes for each of the *n *suffixes in *S*. Each edge in *T *is labeled with a substring of variable length of *S *called an edge-label. Concatenating edge-labels along a path from the root to a node *i *forms a substring, called *i*'s *path-label *in *S*. Leaves in the tree are labeled with the position where the path-label begins in *S*. Internal nodes have at least 2 children, representing positions where repeated substrings diverge. The edge-labels of the children of a node each begin with a different character from the alphabet, so there is at most one child for each letter of the reference string's alphabet. Consequently, the depth of any leaf is at most *n*, and there are *O*(*n*) nodes in the tree.

A suffix tree can be constructed in *O*(*n*) time and *O*(*n*) space for a string over a fixed alphabet, such as for DNA or amino acids, by using additional pointers in the tree called *suffix links*. The suffix link of node *v *with path-label *xα *points to node *v' *with path-label *α *where *x *is a single character and *α *is a substring [13,14]. Suffix links are used to navigate between equivalent nodes of consecutive suffixes without returning to the root of the tree.

All substrings of a query string *Q *of length *m *that occur in a string *S *can be determined in time proportional to *m *by navigating the suffix tree *T *of *S *to follow the characters in *Q*. The algorithm begins by finding the longest prefix of *Q *that occurs in *T*, descending from the root of *T *and following exactly aligning characters in *Q *for as long as possible. Assume that substring *Q*[1, *i*] is found in *T *along the path-label to node *v*, but there is no edge from *v *labeled with the next character in *Q *because *Q*[1, *i *+ 1] is not present in *S*. The algorithm can then report the occurrences of *Q*[1, *i*] at the positions represented by all leaves in the subtree rooted at *v *after checking the alignments are maximal by comparing the left flanking base of the query and reference. The algorithm then continues by finding the longest substrings for each of the *m *- 1 remaining start positions in *Q*. However, instead of navigating the tree from the root each time, the algorithm resumes aligning with *Q*[*i *+ 1] after following the suffix link from *v *to *v' *and without reprocessing previously aligned characters.

**Figure 1:**
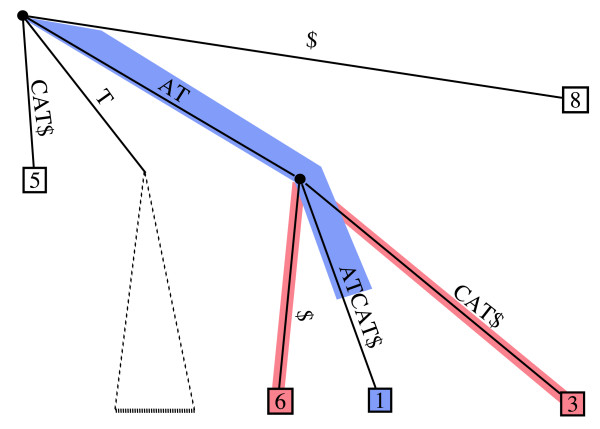
**Aligning a query against a suffix tree**. Aligning the query ATAT against the suffix tree for ATATCAT$. The path from the root to each leaf encodes a sequence that occurs in the reference at the label of that leaf. The blue path shows the extent of the alignment in the tree. The query occurs at position 1 with a alignment length of 4. For *l *≥ 2, MUMmerGPU will print the red nodes as alignments with an alignment length equal to 2, the sequence depth of the lowest common ancestor between the red nodes and the blue node.

Given a user-specifed minimum length *l *and a query *Q*, suppose there is an exact alignment of length *M *≥ *l *for the substring starting at position *i *in the query and ending at or along the edge to node *N*. The length of the alignment (*M*) is equal to the length of the path-label of the parent of node *N *plus the length along the edge to *N*. Starting from *N*, the algorithm follows successive parent links up the tree, subtracting the edge length of each link from the alignment length, until the alignment length is less than *l *as shown in Figure 1. Let *R *be the node with the smallest string depth greater than *l *on this path. For each leaf *L *in the subtree rooted by *R*, the path-label to the lowest common ancestor of *N *and *L *defines a substring starting at *i *in *Q *which occurs in both *Q *and *S *at the reference position defined by the leaf label of *L*. For a thorough discussion of suffix trees and their applications, see Gusfield's classic work on sequence analysis [14].

### GPGPU programming

As the GPU has become increasingly more powerful and ubiquitous, researchers have begun exploring ways to tap its power for non-graphics, or general-purpose (GPGPU) applications [15]. This has proven challenging for a variety of reasons. Traditionally, GPUs have been highly specialized with two distinct classes of graphics stream processors: vertex processors, which compute geometric transformations on meshes, and fragment processors, which shade and illuminate the rasterized products of the vertex processors. The GPUs are organized in a streaming, data-parallel model in which the processors execute the same instructions on multiple data streams simultaneously. Modern GPUs include several (tens to hundreds) of each type of stream processor, so both graphical and GPGPU applications are faced with parallelization challenges [16]. Furthermore, on-chip caches for the processing units on GPUs are very small (often limited to what is needed for texture filtering operations) compared to general purpose processors, which feature caches measured in megabytes. Thus, read and write operations can have very high latency relative to the same operations when performed by a CPU in main memory.

Most GPGPU successes stem from scientific computing or other areas with a homogeneous numerical computational component [17,18]. These applications are well suited for running on graphics hardware because they have high *arithmetic intensity *– the ratio of time spent performing arithmetic to the time spent transferring data to and from memory [19]. In general, the applications that have performed well as a GPGPU application are those that can decompose their problems into highly independent components each having high arithmetic intensity [20]. Some bioinformatics applications with these properties have been successfully ported to graphics hardware. Liu *et al*. implemented the Smith-Waterman local sequence alignment algorithm to run on the nVidia GeForce 6800 GTO and GeForce 7800 GTX, and reported an approximate 16× speedup by computing the alignment score of multiple cells simultaneously [21]. Charalambous *et al*. ported an expensive loop from RAxML, an application for phylogenetic tree construction, and achieved a 1.2× speedup on the nVidia GeForce 5700 LE [22].

**Figure 2:**
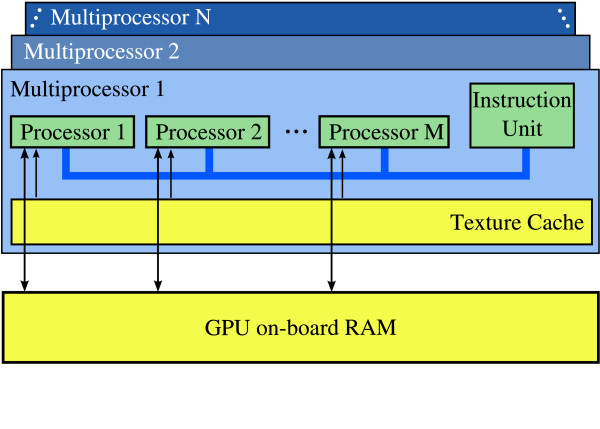
**Simplified view of the nVidia G80 Architecture**. This figure, inspired by a similar figure in [23] shows how the GPU is organized into several (N) multiprocessors, each containing multiple (M) stream processors that simultaneously execute the same instruction. Each processor can access the texture cache very quickly, but reads and writes to the onboard RAM have high latency.

nVidia's new G80 architecture radically departs from the traditional vertex+fragment processor pipeline. It features a set of multiprocessors that each contain a number of stream processors (Figure 2). Graphics applications can use these as either vertex or fragment processors, and GPGPU applications can program them for general computation. All processors on a single multiprocessor simultaneously execute the same instruction, but different multiprocessors can execute different instructions. nVidia anticipated the benefits of such a unified architecture for GPGPU computing, and released the Compute Unified Device Architecture (CUDA) SDK to assist developers in creating non-graphics applications that run on the G80 and future GPUs. CUDA offers improved flexibility over previous GPGPU programming tools, and does not require application writers to recast operations in terms of geometric primitives, as was required by earlier GPGPU environments [23].

**Figure 3:**
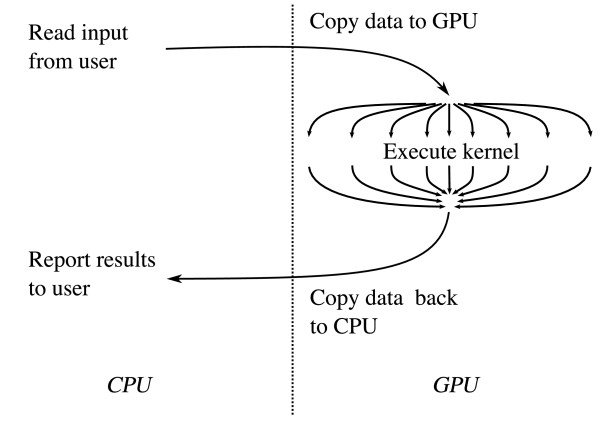
**Typical GPGPU application flow**. Input data for a GPGPU application must be copied to the GPU's memory along with a pre-allocated output buffer prior to invoking the GPU-based kernel. Output from the kernel is read back into main memory and reported to the user.

CUDA enables programmers to write programs that run on the GPU in a restricted form of the C programming language, and compiled into G80 bytecode. CUDA programs typically consist of a component that runs on the CPU, or *host*, and a smaller but computationally intensive component called the *kernel *that runs in parallel on the GPU (Figure 3). The kernel cannot access the CPU's main memory directly – input data for the kernel must be copied to the GPU's on-board memory prior to invoking the kernel, and output data also must first be written to the GPU's memory. All memory used by the kernel must be preallocated, and the kernel cannot use recursion or other features requiring a stack, but loops and conditionals are allowed. Furthermore, the number of registers per multiprocessor is limited and the multiprocessor schedules fewer processors to compute simultaneously if the number of registers used per kernel is too high. Consequently, high-performance kernel code requires careful tuning to reduce the number of registers used and limit the amount of branching.

The improved flexibility of CUDA does not solve the more fundamental problems caused by the G80's stream-computing organization: the relatively small cache and associated high memory latency for memory intensive programs. However, the G80's texture memory is cached to speed up memory intensive texture mapping operations, and can be used by GPGPU programs. GPGPU programs can pack their data structures into one-, two-, or three-dimensional arrays stored in texture memory, and thus use the cache for read-only memory accesses to these data structures [23]. Performance is further improved by utilizing one of several software techniques for maximizing the benefit offered by even a small cache. One such class of techniques involves reordering either the data in memory or the operations on those data to maximize data and temporal locality. Mellor-Crummey *et al*. reported significant speedup in particle interaction simulations, which feature highly irregular access patterns, by reordering both the locations of particles in memory and the order in which interactions were processed. They tested a reordering strategy based on space-filling curves, such as the Hilbert and Morton curves [24].

## Implementation

**Figure 4:**
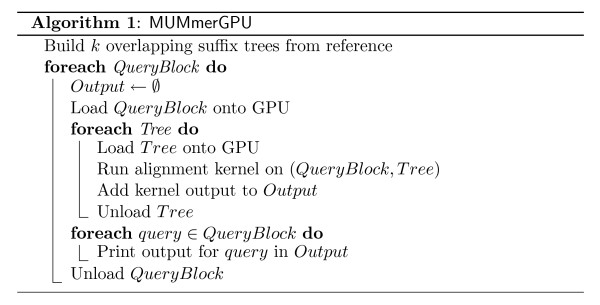
**MUMmerGPU Algorithm**. MUMmerGPU builds multiple suffix trees of the reference and partitions the query sequences into sets, called *QueryBlocks*, depending on the memory available on the GPU. Sequences within a given *QueryBlock *are aligned in parallel on the GPU.

The MUMmerGPU algorithm performs parallelized exact string alignment on the GPU (Figure 4). First a suffix tree of the reference sequence is constructed on the CPU using Ukkonen's algorithm [13] and transfered to the GPU. Then the query sequences are transfered to the GPU, and are aligned to the tree on the GPU using the alignment algorithm described above. Alignment results are temporarily written to the GPU's memory, and then transfered in bulk to host RAM once the alignment kernel is complete for all queries. Finally, all maximal alignments longer than a user-supplied value (*l*) are reported by post-processing the raw alignment results on the CPU. The output format and many parameters of MUMmerGPU are identical to those of mummer (with the -maxmatch option), up to the order in which alignments appear in the output for each query, and thus MUMmerGPU can be used as a drop-in replacement for mummer. In particular, all programs in the NUCmer suite of programs that use the output of mummer, including those that extend the exact alignment seeds to larger inexact alignments, can take advantage of the GPU paralellization [4-6].

The G80 has a relatively small amount of on-board memory, so the data are partitioned into large blocks so that the reference suffix tree, query sequences, and output buffers will fit on the GPU. As of this writing, the amount of on-board memory for a G80 ranges from 256 MB to 768 MB. A suffix tree built from a large reference sequence, such as a human chromosome, will exceed this size, so MUMmerGPU builds *k *smaller suffix trees from overlapping segments of the reference. MUMmerGPU computes *k *at runtime to fill approximately one third of the total GPU device memory with tree data. The trees overlap in the reference sequence by the maximum query length *m *supported by MUMmerGPU (currently 8192 bp) to guarantee all alignments in the reference are found, but alignments in the overlapping regions are reported only once.

After building the trees, MUMmerGPU computes the amount of GPU memory available for storing query data and alignment results. The queries are read from disk in blocks that will fill the remaining memory, concatenated into a single large buffer (separated by null characters), and transferred to the GPU. An auxiliary 1D array, also transfered to the GPU, stores the offset of each query in the query buffer. Each multiprocessor on the GPU is assigned a subset of queries to process in parallel, depending on the number of multiprocessors and processors available. The executable code running on each processor, the *kernel*, aligns a single query sequence from the multiprocessor's subset to the reference. The kernel aligns the query to the reference by navigating the tree using the suffix-links to avoid reprocessing the same character of the query, as described above. Reverse complement alignments are computed using a second version of the kernel which reverse complements the query sequences on-the-fly while aligning, allowing for computing both forward and reverse alignments without any additional data transfer overhead. The output buffer contains a slot to record the alignment result for each of the *m *- *l *+ 1 substrings for a query of length *m*. The fixed size alignment result consists of the node id of the last visited node in the tree and length of the substring that exactly aligns. This information is sufficient to print all positions in the reference that exactly align the substring on the CPU.

After the kernel is complete for all the queries, the output buffer on the GPU is transfered to host RAM and the alignments are printed by the CPU. Each slot in the output buffer corresponds to a specific substring of a query. If multiple trees were built from the reference (*k *> 1), then the output slots for each tree are preserved until the queries in a block have been aligned against each tree. This way all of the alignments for a given query can be printed in a single block, following the syntax used by mummer.

### GPU Memory Layout

The suffix tree is "flattened" into two 2D textures, the node texture and the child texture. Each tree node is stored in a pair of 16-byte texels (texture elements) in these two textures. The node texture stores half the information for a node, including the start and end coordinates of the edge sequence in the reference, and the suffix link for the node. The remaining information for a node – the pointers to its A, C, G & T children – is stored in the child texture, addressed in parallel to the node texture. An auxiliary table containing each node's edge length, sequence depth, parent pointer, and suffix number for leaf nodes, is stored in RAM and is used during the output phase.

**Figure 5:**
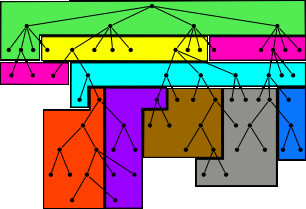
**Suffix Tree layout**. The nodes of the suffix tree are rearranged into cache blocks to optimize 2D locality. Near the root of the tree, nodes of the same depth are placed into the same "wide" block. Futher down the tree, nodes from the same subtree are placed into the same "tall" block. MUMmerGPU uses blocks of 32 × 32 nodes, but for clarity, 8 nodes cache blocks are displayed here.

In the CUDA architecture, a program can store read-only data as cached textures. The G80's proprietary caching scheme takes advantage of 2D locality common in texturing operations. Therefore, the algorithm attempts to optimize the 2D locality of the tree structure in these textures by organizing the nodes in 32 × 32 texel blocks as shown in Figure 5. Near the root of the tree (node depth *<*16), nodes are assigned using a level-order (breadth-first) traversal of the tree creating "wide" blocks of the tree. This ensures that all nodes near the root of the tree are placed in the first 32 × 32 texel blocks, and guarantees the children of a given node will be at (nearly) adjacent cells in the texture. This is useful because at this depth, loading a single 32 × 32 block for one kernel is likely to be reused for the other kernels running in parallel. Further from the root (depth ≥ 16), nodes are arranged in "tall" blocks so that a node, its children, grandchildren, and great-grandchildren are adjacently placed in the same (or adjacent) 32 × 32 block. As multiple queries are aligned against lower parts of the tree, it becomes less likely that their kernels will access many of the same nodes. Thus, the data reordering scheme attempts to increase the cache hit rate for a single thread. The exact specification of the G80's caching scheme is proprietary information, but empirically, this hybrid layout seems to maximize the cache hit rate near the root of the tree, and towards the leaves where the kernel access patterns are radically different.

The reference sequence for the tree is transferred to the GPU as a third 2D texture, and is reordered along a simple 2D space-filling curve to maximize the cache hit rate for subsequent accesses along a node's edge. The sequence is reordered so that beginning with the first character, every four characters in the reference become the topmost four characters in the columns of the 2D array. Once the array contains 4 × 65,536 characters, successive four-character chunks become the next four characters in the columns, left-to-right, and so on. We experimented with a variety of other data reordering schemes, including along a Morton curve and other space filling curves, and found this to have the best performance on several reference sequences. Altogether, using cache memory organized with the spacing-filing curves for the suffix tree and reference sequence improved the kernel execution speed by several fold.

### Complexity of MUMmerGPU

MUMmerGPU constructs its suffix trees in *O*(*n*) time with Ukkonen's algorithm, where *n *is the length of the reference. The alignment kernel running on the card computes all exact substring alignments for each query in time linear in the length of the query. The kernel is an implementation of existing alignment methods [14], but with many independent instances running simultaneously on the GPU.

MUMmerGPU uses both GPU memory and main system memory. Suffix trees use an amount of memory linear in the length of the reference from which they are constructed [14]. The suffix trees in MUMmerGPU thus each occupy *O*(*n/k *+ *m*) space, where *k *is the number of overlapping trees specified by the user, and *m *is the maximum query length supported by MUMmerGPU. Note that for most expected uses of MUMmerGPU *n *≫ *m*. Only a fraction of that total space is actually transferred to the GPU. In the current implementation, 32 out of every 48 bytes per node are transferred. The remaining bytes are stored in the host-only auxiliary table used only for printing results by the CPU. For each query, MUMmerGPU transfers the null terminated query sequence prepended with a special mismatch character, along with two 4-byte entries in auxiliary tables used by the kernel. For a query of length *m*, and a minimum substring length *l*, *m *- *l *+ 1 output slots are reserved to record the query's substring alignments, and each output slot occupies 8 bytes. The total space required on both the CPU and the GPU for each query is 8(*m *- *l *+ 1) + (*m *+ 10) bytes. On a G80 with 768 MB of on-board RAM, there is sufficient RAM to store a tree for a 5 Mbp reference sequence, and 5 million 25 bp or 500,000 100 bp query sequences.

## Results and Discussion

We measured the relative performance of MUMmerGPU by comparing the execution time of the GPU and CPU version of the alignment code, and the total application runtime of MUMmerGPU versus the serial application mummer. The test machine has a 3.0 GHz dual-core Intel Xeon 5160 with 2 GB of RAM, and an nVidia GeForce 8800 GTX. The 8800 GTX has 768 MB of on-board RAM and a G80 with 16 multiprocessors, each of which has 8 stream processors. At the time of this writing, the retail price of the 8800 GTX card is $529, and a retail-boxed Intel Xeon 5160 CPU is $882 [25]. Input and output was to a local 15,000 RPM SATA disk. The machine was running Red Hat Enterprise Linux release 4 update 5 (32 bit), CUDA 1.0, and mummer 3.19.

We ported the MUMmerGPU alignment kernel to use the CPU instead of the GPU to isolate the benefit of using graphics hardware over running the same algorithm on the CPU. CUDA allows programmers to write in a variant of C, so porting MUMmerGPU to the CPU required only straightforward syntactic changes, and involved no algorithmic changes. Where the CUDA runtime invokes many instances of the kernel on the GPU simultaneously, the CPU executes each query in the block sequentially.

The first test scenario was to align synthetically constructed reads to a bacterial genome. We used synthetic reads in order to explore MUMmerGPU's performance in the absence of errors and over a wider variety of query lengths then are available with genuine reads. The synthetic test reads consisted of 50-, 100-, 200-, 400-, and 800-character substrings (uniformly randomly) sampled from the *Bacillus anthracis *genome (GenBank ID: NC_003997.3). Thus, each read exactly aligns to the genome end-to-end at least once, and possibly more depending on the repeat content of the genome. When aligning each of the five sets of reads, we used *l *equal to the read size for the set. Each set contained exactly 250,000,000 base pairs of query sequence divided evenly among all the reads in the set.

**Figure 6:**
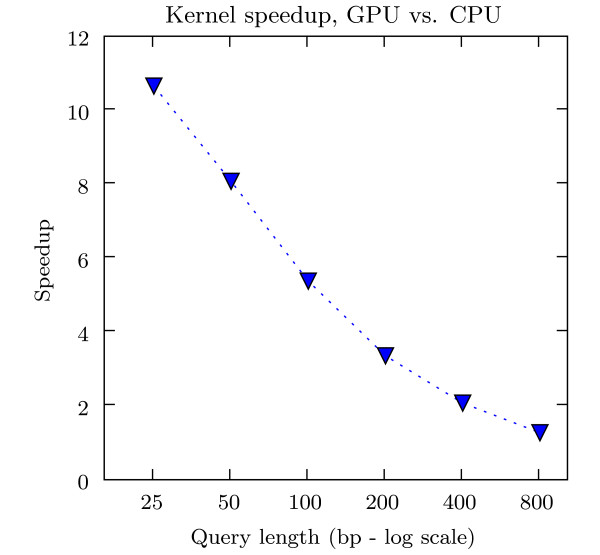
**Speedup of MUMmerGPU on the GPU over the CPU**. The decrease in speedup when processing error-free synthetic reads as read length increases is due to a combination of thread divergence and poor cache hit rate.

The time for building the suffix tree, reading queries from disk, and printing alignment output is the same regardless of whether MUMmerGPU ran on the CPU or the GPU, since those parts of MUMmerGPU always run on the CPU. The actual sequence alignment portion of MUMmerGPU ran dramatically faster, over 10× faster, on the GPU, despite the added cost of transferring the tree and query data to the GPU. The speedup of MUMmerGPU (not including the costs mentioned above shared by both variants) running on the GPU over MUMmerGPU on the CPU is shown in Figure 6.

For longer reads, the speedup of using the GPU is diminished, because of poor cache performance and thread divergence, both of which are acknowledged as potential performance problems on the G80 [23]. All queries begin at the root of the tree, and many queries will share common nodes on their paths in the tree. However, as the kernel travels deeper into the tree for longer reads, the texture elements stored in the cache are reused less often, thus reducing the cache hit rate, and increasing the overall average access time. In addition, even though queries are the same length, the alignment kernel may not visit the same number of nodes, nor spend the same amount of time comparing to edges, because edges in suffix trees have variable length. This creates divergence among the threads processing queries, and the multiprocessor will be forced to serialize their instruction streams. It is difficult to quantify the relative contribution of these effects, but it is likely that both are significant sources of performance loss.

**Figure 7:**
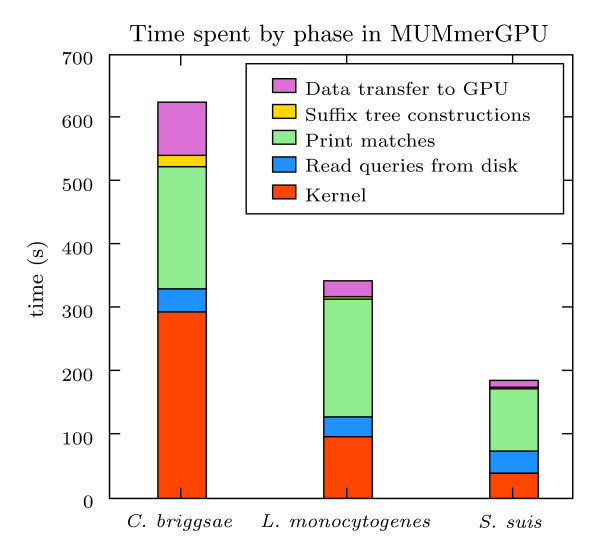
**Breakdown of MUMmerGPU processing time**. The stacked bar charts indicate the amount of time spent in each phase of the MUMmerGPU for the three test sets. Given a sufficiently large number of sequencing reads, the time spent building the suffix tree is small compared to time spent aligning queries.

In addition to the test with synthetic data, we also aligned reads from several recent sequencing projects against the genomes from which the reads were generated. The projects included *Streptococcus suis *sequenced with the Solexa/Illumina sequencer [26], multiple strains of *Listeria monocytogenes *sequenced using 454 pyrosequencing (Genome GenBank ID: NC_003210.1, read TI numbers 1405533909 – 1405634798, 1406562010 – 1406781638, 1407073020 – 1411183505, 1413490052 – 1415592095, 1415816363 – 1415903784) and *Caenorhabditis briggsae *sequenced with standard ABI 3730xl Sanger-type sequencing [27]. We aligned the reads against both strands of the chromosomal DNA for *L. monocytogenes *and *S. suis*, and against both strands of chromosome III of *C. briggsae*. Little data from Solexa/Illumina has been made public at the time of this writing, and the public data set available had only a single lane's worth of data. To represent the full set of reads from a full Solexa/Illumina run, we concatenated 10 copies of a publicly-available file containing 2,659,250 36 bp reads to form the *S. suis *query set. The reference sequence and queries in all three tests did not include ambiguous bases. For these three tasks, Table 1 shows the runtime parameters used and the overall speedup of MUMmerGPU over mummer. Figure 7 shows the wall-clock time spent by MUMmerGPU in the various phases of the algorithm, including kernel execution and I/O between CPU and GPU.

**Table 1:** Runtime parameters and speedup for MUMmerGPU test workloads. MUMmerGPU is consistently more than 3 times faster than mummer for a variety of sequencing data.

Reference	Reference length (bp)	# of queries	Query length mean ± stdev.	Min alignment length (*l*)	# of suffix trees (*k*)	Speedup
*C. briggsae Chr. III *(Sanger)	13,163,117	2,357,666	717.84 ± 159.44	100	2	3.71
*L. monocytogenes *(454)	2,944,528	6,620,471	200.54 ± 60.51	20	1	3.79
*S. suis *(Illumina/Solexa)	2,007,491	26,592,500	35.96 ± 0.27	20	1	3.47

For each of the alignment tasks, MUMmerGPU was between 3.47 and 3.79 times faster than mummer. For *C. briggsae*, MUMmerGPU spent most of its time aligning queries on the GPU. Because we aligned all of the reads from the sequence project against chromosome III of the *C. briggsae*, many of the reads did not align anywhere in the reference. As a result, a relatively short amount of time was spent in writing alignment output to disk. For other alignments, such as for the *L. monocytogenes *and *S. suis *test sets, the output phase dominates the running time of MUMmerGPU. For these tasks, printing the output in parallel with aligning a block of queries would provide substantial speedup, as it would hide much of the time spent aligning queries on the card. We plan to adopt this strategy in a future release of MUMmerGPU.

Despite the performance hazards experienced for longer simulated reads, MUMmerGPU on the GPU consistently outperforms mummer on real sequencing data by more than a factor of three in wall-clock application running time. Unlike the idealized simulated reads, these reads are variable length and have sequencing error, which will cause further divergence in the kernel executions. Furthermore, the *C. briggsae *alignment required the use of a segmented suffix tree and associated data transfer overhead. In general, MUMmerGPU confers significant speedup over mummer on tasks in which many short queries are aligned to a single long reference.

## Conclusion

Operations on the suffix tree have extremely low arithmetic intensity – they consist mostly of following a series of pointers. Thus, sequence alignment with a suffix tree might be expected to be a poor candidate for a parallel GPGPU application. However, our results show that a significant speedup, as much as a 10-fold speedup, can be achieved through the use of cached texture memory and data reordering to improve access locality. This speedup is realized only for large sets of short queries, but these read characteristics are beginning to dominate the marketplace for genome sequencing. For example Solexa/Illumina sequencing machines create on the order of 20 million 50 bp reads in a single run. For a single human genotyping application, reads from a few such runs need to be aligned against the entire human reference genome. Thus our application should perform extremely well on workloads commonly found in the near future. The success of our application is in large part the result of the first truly general purpose GPU programming environment, CUDA, which allowed us to directly formulate and implement our algorithm in terms of suffix tree navigation and not geometric or graphics operations. This environment made it possible to efficiently utilize the highly parallel and high speed 8800 GTX. An 8800 GTX is similar in price to a single 3.0 Ghz Xeon core, but offers up to 3.79× speedup in total application runtime. Furthermore, in the near future, a common commodity workstation is likely to contain a CUDA compliant GPU that could be used without any additional cost.

Even though MUMmerGPU is a low arithmetic memory intensive program, and the size of the stream processor cache on the G80 is limited, MUMmerGPU achieved a significant speedup, in part, by reordering the nodes to match the access patterns and fully use the cache. We therefore expect with careful analysis of the access pattern, essentially any highly parallel algorithm to perform extremely well on a relatively inexpensive GPU, and anticipate widespread use of GPGPU and other highly parallel multicore technologies in the near future. We hope by making MUMmerGPU available open source, it will act as a roadmap for a wide class of bioinformatics algorithms for multi-processor environments.

## Availability and requirements

Project name: MUMmerGPU

Project home page: 

Operating system(s): Linux, UNIX

Programming language: C, C++, CUDA

Other requirements: nVidia G80 GPU, CUDA 1.0

License: Artistic License

Restrictions to use by non-academics: none.

## Authors' contributions

MS and CT developed the software and wrote the manuscript together. AD and AV helped to draft and edit the manuscript. All authors read and approved the final manuscript.
